# Sex Differences in the Effects of Mental Work and Moderate-Intensity Physical Activity on Energy Intake in Young Adults

**DOI:** 10.5402/2013/723250

**Published:** 2013-05-29

**Authors:** Emilie Pérusse-Lachance, Patrice Brassard, Jean-Philippe Chaput, Vicky Drapeau, Normand Teasdale, Caroline Sénécal, Angelo Tremblay

**Affiliations:** ^1^Department of Physical Activity Sciences, University of Quebec, Trois-Rivieres, QC, Canada G9A 5H7; ^2^Department of Kinesiology, Faculty of Medicine, Laval University, Quebec City, QC, Canada G1V 0A6; ^3^Healthy Active Living and Obesity Research Group, Children's Hospital of Eastern Ontario Research Institute, Ottawa, ON, Canada K1H 8L1; ^4^Department of Physical Education, Faculty of Education, Laval University, Quebec City, QC, Canada G1V 0A6; ^5^School of Psychology, Faculty of Social Sciences, Laval University, Quebec City, QC, Canada G1V 0A6

## Abstract

The aim of this study was to examine the acute effects of mental work and moderate-intensity physical activity on various components of energy balance in young and healthy adults. With the use of a randomized crossover design, 35 participants aged 24 ± 3 years completed three 45-min conditions, namely, (i) resting in a sitting position (control), (ii) reading and writing (mental work (MW)), and (iii) exercising on a treadmill at 40% of peak oxygen uptake (exercise), followed by an *ad libitum* lunch. The endpoints were spontaneous energy intake (EI), energy expenditure (EE), appetite sensations, and EI for the remainder of the day. We observed that the energy cost of the control and MW conditions was about the same whereas the exercise condition increased EE to a greater extent in men than women. Exercise induced a decrease in EI relative to EE compared to the control condition that was more pronounced in men than women. However, women tended to increase their energy intake after the MW condition compared to the control one whereas an opposite trend was observed in men. None of the appetite sensation markers differed significantly between both sexes. In conclusion, men and women have specific food intake patterns when submitted to cognitive and physical stimuli.

## 1. Introduction

The obesity epidemic that has been described by the World Health Organization (WHO) [1] is more prevalent in women than in men [2]. Despite this greater proneness of women to accumulate body fat when exposed to an obesogenic environment, women are less affected by obesity comorbidities because of difference in fat distribution. Specifically, their predisposition to accumulate an excess body fat in the gluteo-femoral area as opposed to the abdominal area for men has frequently been proposed as less detrimental for health or even, in some cases, as a protective storage strategy [3]. 

The study of potential sex differences has also been performed regarding the response of energy balance to different lifestyle modalities. For instance, it has been established several decades ago that body weight changes induced by exercise training are more pronounced in male than in female rats [4, 5]. In humans, our research team obtained concordant results by demonstrating a significant weight loss induced by exercise training in men whereas no significant changes were found in women [6]. In addition, preprandial caffeine consumption resulted in a significant decrease in *ad libitum* energy intake in men but not in women [7]. More recently, the response of energy compensation to vigorous physical activity was tested under standardized laboratory conditions. In the Imbeault et al. (1997) study, vigorous exercise induced a significant decrease in spontaneous postexercise energy intake relative to energy expenditure in men compared to a control condition in a relaxing seated position [8]. In contrast, Pomerleau et al. (2004) reported an increase in postexercise energy intake in obese women tested under comparable laboratory conditions [9]. The literature about energy compensation after exercise is controversial and highly variable [10, 11], and this would justify the reexamination of the effect of exercise on energy intake. 

The sex dimorphism in the response to exercise is likely to be observed when one is exposed to other activities or stimuli under free-living conditions. In this regard, we have recently demonstrated that challenging cognitive effort increases *ad libitum* energy intake, even when hunger is not significantly increased [12, 13]. This effect might be related to the increase in plasma cortisol and glucose instability that were also found to be promoted by mental work [12, 14, 15]. Up to now, data that we reported about the impact of cognitive effort on energy intake and feeding behaviour were obtained in women. However, on the basis of the literature described above which emphasises the existence of sex differences in response to physical activity, we believe that the same phenomenon might exist when one is exposed to the mental work stimulus. 

Therefore, the main aim of this study was to evaluate the response of energy intake and appetite sensations of young men and women to a session of mental work having been shown to favour hyperphagia [12, 13]. In addition, we took advantage of this protocol to reexamine the issue of sex differences in the response of energy intake and appetite sensations to standardized exercise testing.

## 2. Methods

### 2.1. Subjects and Procedures

Thirty-five young adults (22 men and 13 women), aged 24 ± 3 years, participated in this crossover study. Because it is known that spontaneous energy intake significantly differs between the follicular and the luteal phase of the cycle, women participants were tested within day 0 and 10 of their menses in order to minimize the influence of women's reproductive hormones [16]. To be considered in this study, subjects had to be nonsmokers, to have no hearing problems, to have no cardiovascular disease, to have no metabolic problem and/or not to take medication that could interfere with the objectives of the study, to be free of eating disorders, to have a stable body weight since 6 months (±2 kg), to have a body mass index between 20 and 30 kg/m^2^, to be nonvegetarian or nonvegan, not to be pregnant or to have an irregular menstrual cycle, to be free of food allergies, not to be a restrained eater, that is, having a score >12 for cognitive dietary restraint in the Three-Factor Eating Questionnaire (TFEQ), to be physically inactive or moderately active, that is, <3 hours of physical activity per week, and to be familiar with the use of a computer.

Before each testing day, the subjects followed a standardized protocol including the following guidelines: (1) no caffeine and/or alcohol 24 h before the test, (2) no physical activity 24 h before the test, and (3) a standardized breakfast (women: 598 kcal, men: 714 kcal) 3 h before the test after a 12-h overnight fast. The standardized breakfast consisted of white bread (women: 80 g and men: 100 g), butter (women: 12 g, men: 12 g), peanut butter (women: 16 g, men: 16 g), cheddar cheese (women: 20 g, men: 40 g), and orange juice (women: 200 mL, men: 200 mL). The subjects were blinded in regard of the main hypothesis of the study. All subjects gave their written consent, prior to their inclusion, to participate in this study, which received the approval of the Laval University ethics committee. 

### 2.2. Study Protocol

All participants were randomly assigned to three experimental conditions lasting 45 min: (i) control condition, consisting in a resting period comfortably seated in an arm chair, subjects were not allowed to talk, to sleep, to read, or to watch TV, (ii) a reading-writing condition, consisting in reading a 10-page text and writing a summary of approximately 350 words using a computer (mental work), and (iii) exercising (walking) on a treadmill at 40% of maximal oxygen uptake (exercise), followed by an *ad libitum* lunch. These three experimental conditions were randomly assigned by using a computerized randomization scheme. The participants were tested one at a time, on 3 different occasions, 1–4 weeks apart.

### 2.3. Visual Analogue Scales (VAS)

All subjects had to fill seven VAS at the beginning (T-60/60 minutes before the buffet), after the experimental session (T-15/15 minutes before the buffet), and after the buffet-type meal (T0, T60, T120, T180, and T240) to assess appetite sensations. VAS at T60, T120, T180, and T240 were assessed outside of the laboratory, and participants were not allowed to eat and/or to drink and/or to practice high-intensity physical activity during those 4 hours. Desire to eat, hunger, fullness, prospective food consumption, and desire to eat salty, sweet, or fatty foods were rated on 150 mm VAS. Methods regarding VAS measurement have been previously described by Doucet et al. [17], and VAS measurements have been recognized for being highly reproducible [18]. 

### 2.4. Buffet-Type Lunch and Dietary Record

*Ad libitum* food intake was measured by following the methodology previously described by Arvaniti et al. [18]. The subject was offered a cold buffet-type meal which included a wide variety of foods after each condition (control, mental work, and exercise). All participants were asked to eat *ad libitum* in a 30 min period. All foods were weighed before and after the meal to accurately quantify food intake and participants were blinded to this assessment. After each condition, subjects had to fill out a 36-hr dietary record in order to assess the degree of compensation in food intake following the condition. Mean energy and macronutrient intakes were estimated with a computerized version of the Canadian Nutrient File by a dietician [19].

### 2.5. Relative Energy Intake

In order to get a proxy value of short-term variations in energy balance, we calculated the relative energy intake (REI), which corresponds to energy intake during the buffet-type meal from which we subtracted the energy cost of each experimental condition above resting level. Resting energy expenditure was measured on a breath-by-breath basis using an automated gas analyzing system (K^4^B_2_ by Cosmed) during the control condition. For each experimental condition, the same measure technique was used to assess the energy cost. Relative energy intake was then calculated as follows:

(1) REI (kcal) =Energy intake (kcal)  −[(Energy cost in each condition (kcal/min⁡)    −Resting energy expenditure (kcal/min⁡))   ∗45 min⁡].

### 2.6. Exercise Intensity

Peak oxygen uptake (VO_2_ peak) was measured at least 2 days before the beginning of the protocol. Expired air was continuously collected for the determination of VO_2_ on a breath-by-breath basis using an automated gas analysing system (K^4^B_2_ by Cosmed). The maximal test protocol consisted of walking and running on a treadmill with an increasing work output to the point of exhaustion. VO_2_ peak was defined as the mean VO_2_ recorded in the last 20 seconds of the incremental exercise protocol concomitant to a respiratory exchange ratio (RER) ≥ 1.0. The exercise protocol was always performed at the same time of the day at 23°C room temperature. Then, the intensity of treadmill exercise was fixed at 40% VO_2_ peak for the exercise condition.

### 2.7. Statistical Analysis

A two-way analysis of variance for repeated measures was performed on the means of all variables. A Tukey HSD post hoc test was then performed in order to contrast mean differences between the mental work and the exercise condition (multiple comparison with Bonferroni's correction). The statistical significance was set at a *P* value < 0.05. All statistical analyses were performed using the JMP version 8.0.1 (SAS Institute, Cary, NC).

## 3. Results

Thirty-five subjects participated in this study (22 men and 13 women) and were aged 24 ± 3 years. They were all healthy and had a normal weight. None of the participants were athletes and/or had eating disorders.  Table 1 presents the descriptive characteristics of all participants.

Table 2 presents data on the energy cost and relative energy intake (REI) of each experimental condition between sexes. As expected, energy expenditure during the mental work and exercise sessions was lower in women than in men (*P* < 0.0001). The energy cost of the mental work session was not different from the control condition in both sexes. The difference between REI after the mental work and the control sessions was higher in women than men (women: +101 ± 270 kcal versus men: −210 ± 310 kcal; *P* < 0.05), and the same pattern was observed for the difference between REI after the exercise and the control sessions (women: −159 ± 243 kcal versus men: −433 ± 344 kcal; *P* < 0.05).

**Figure 1:**
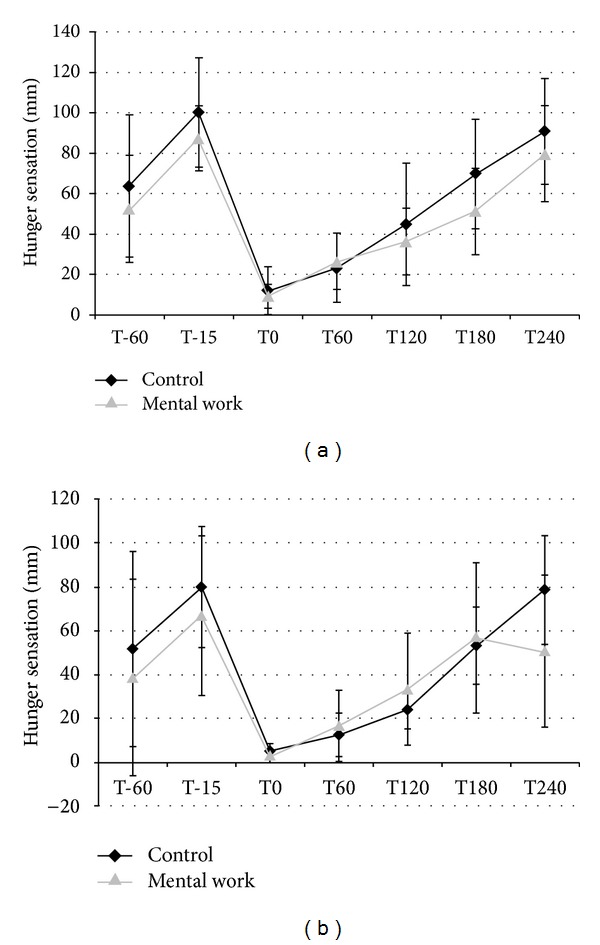
Hunger sensation for men (a) and women (b) during the mental work and the control conditions. All data are not statistically significant. T0 = immediately after the buffet-type meal.

Figure 1 shows that no differences were present in hunger appetite sensation between the mental work and the control condition in both sexes. As observed in Figure 1(a), men had a higher level of hunger before and after the mental work condition. The buffet-type meal induced a reduction of hunger which increased gradually during the subsequent hours. These changes were not statistically significant. The same pattern was shown in women (Figure 1(b)), and no difference was observed between the control and the mental work conditions. The exercise condition exerts the same effect on hunger appetite sensation in both men and women likewise not to a statistical standpoint. The other appetite sensations that were measured followed the same pattern as the hunger appetite sensation, and changes were not statistically significant (results not shown).

Finally, Table 3 presents the mean energy intake for the remainder day assessed by a 36-h dietary record. Compensation was observed in food intake in the three conditions neither in men nor women. However, in each condition, women consumed fewer calories for the remainder of the day than men.

## 4. Discussion

The main purpose of this study was to assess the response of energy intake and appetite sensations to mental work in men and women. We also examined the possibility of sex differences in energy intake and appetite sensations exercise. Our results suggest that an experimental mental work condition consisting of reading and computer writing a brief document may affect differently food intake in men and women without affecting appetite sensations. In addition, these results support the idea of a sex difference in energy intake following a 45 min period of moderate-intensity physical activity in a laboratory context. Globally, the results of this study show that food intake adapts in response to these two stimuli in a way that is more susceptible to prevent a negative energy balance in women than in men.

Some investigations have documented the impact of demanding mental work on energy and macronutrient intake. To our knowledge, this issue was examined for the first time by McCann et al. [20] who assessed food intake in scientists from the University of Washington at the time they were actively engaged in the preparation of grant applications. Their results revealed a significant increase in energy and lipid intake that was accompanied by an increase in cholesterolemia. These observations are concordant with the results reported by Wallis and Hetherington [21] who found a significant increase in spontaneous chocolate consumption in subjects exposed to a cognitive effort. Our research team has also been involved in the investigation of this issue. In female students of Laval University, we have demonstrated that a session of reading and computer writing induced a significant increase in *ad libitum* energy intake during a buffet-type meal which followed the testing session [12, 13]. Interestingly, this increase in energy intake occurred without significant changes in hunger and other appetite sensations. Our studies have also enabled us to demonstrate that the apparent hyperphagic effect of mental work is associated with glycemia instability [12, 22] and an increase in plasma cortisol [12]. The results reported in this study are consistent with our previous findings since our female subjects also displayed an increase in energy intake without significant change in appetite sensations in response to the mental work condition. However, this effect was not observed in men who rather exhibited a decrease in energy intake in response to the cognitive effort. 

The sex difference in energy intake observed in response to mental work in the present study agrees with our research experience with the use of other stimuli to influence energy intake and balance in men and women. Indeed, many years ago, we found a greater weight loss in young men than women in response to exercise training [6]. This is concordant with the early literature that was documenting sex differences in the response of energy intake to exercise in animals [23]. Accordingly, a subsequent study using preprandial caffeine supplementation to influence energy intake also revealed the existence of a sex dimorphism in the response of energy intake. Specifically, preprandial caffeine intake reduced *ad libitum* energy intake in men during the subsequent meal by 227 kcal whereas a small increase was noted in women [7]. This is also in agreement with the response to moderate-intensity exercise noted in the present study since relative energy intake decreased to a greater extent in men than in women. 

The use of visual analog scales (VAS) in the present study did not permit to detect any significant change in appetite sensations, be it in response to mental work or exercise. On the basis of our research experience, it is plausible that VAS were used in this study under conditions that did not really permit an adequate discrimination by this type of tool [12]. Indeed, even if we demonstrated that the use of VAS provides reproducible results [18], we have only been able to document a clinical discrimination with VAS in the context of intervention where body weight loss was substantial [24, 25]. Beyond technical considerations, we cannot exclude the possibility that acute changes in energy intake following exercise and mental work can occur without concordant changes in hunger and other appetite sensations.

One of the limitations of this study is the small sample size and its homogeneity which limits generalization of these results to the whole population. However, data were obtained on both men and women, and a number of comparisons turned out to be significant which confirms that these observations deserve importance. Moreover, a large interindividual variability was observed as highlighted by large standard deviation values in appetite sensations markers. The latter could demonstrate that there may be some good and bad responders to mental work and exercise as shown by King et al. [10]. In addition, the menstrual cycle is known to influence energy intake and energy expenditure in women [26]. Accordingly, women tested in this study were evaluated within day 0 and 10 of their menses in order to minimize the influence of women's reproductive hormones. Finally, the pilot nature of our study does not permit to solve the entire question posed at the beginning of this paper, and further research is needed to uncover the underlying mechanisms that could explain such differences in energy intake induced by mental work and moderate-intensity exercise between men and women.

In summary, the results of this study show the existence of a sex difference in the response of energy intake to mental work. They also confirm that the response of women to exercise is more susceptible to prevent a negative energy balance compared to men. This study also shows that variations in energy intake happened without changes in appetite sensations and energy compensation in both men and women.

## Figures and Tables

**Table 1:** Descriptive characteristics of the participants.

	All (*n* = 35)	Men (*n* = 22)	Women (*n* = 13)
Age (years)	24 ± 3	25 ± 3	23 ± 3
Height (cm)	174 ± 9	180 ± 5	165 ± 6**
Body weight (kg)	70 ± 12	76 ± 9	60 ± 7**
BMI (kg/m^2^)	23 ± 2	23 ± 2	22 ± 2
Waist circumference (cm)	80 ± 7	83 ± 6	75 ± 6*
VO_2_ peak (mlO_2_/kg/min)	56.8 ± 10.4	61.7 ± 9.0	47.9 ± 5.8**

**P < 0.0001 between men and women; *P < 0.05 between men and women.

**Table 2:** Energy cost of testing sessions and relative energy intake (REI) in men and women.

	Control		Mental work		Exercise	
	Men	Women	Men	Women	Men	Women
Energy expenditure (kcal)	57 ± 7	45 ± 5**	69 ± 8	54 ± 7**	420 ± 60	259 ± 41**
Energy intake (kcal)	1655 ± 384	789 ± 296**	1399 ± 331	919 ± 343**	1508 ± 374	833 ± 303**
Relative energy intake (kcal)	1655 ± 384	789 ± 296**	**1388 ± 331** ^~^	910 ± 343*	**1139 ± 372** ^ #^	605 ± 329**

**P < 0.0001 between men and women; *P < 0.001 between men and women; ^#^ *P* < 0.05 between mental work and exercise relative energy intake in men; ^~^ *P* < 0.05 between control and mental work relative energy intake in men; data corrected for BMI; REI = energy intake − (energy cost in each condition − resting energy expenditure) ∗ 45 min.

**Table 3:** Mean energy intake for the remainder of the day in each experimental condition.

Energy intake	Control		Mental work		Exercise	
	Men	Women	Men	Women	Men	Women
kcal	4474 ± 1439	2738 ± 569*	4493 ± 1254	2811 ± 539*	4625 ± 1349	2794 ± 812*

*P < 0.001 between men and women in each of the experimental conditions.
